# Canavan’s spongiform leukodystrophy (Aspartoacylase deficiency) with emphasis on sonographic features in infancy: description of a case report and review of the literature

**DOI:** 10.1007/s40477-022-00667-2

**Published:** 2022-02-20

**Authors:** Leon Rossler, Stefan Lemburg, Almut Weitkämper, Charlotte Thiels, Sabine Hoffjan, Huu Phuc Nguyen, Thomas Lücke, Christoph M. Heyer

**Affiliations:** 1 Klinik für Kinder- und Jugendmedizin der Ruhr-Universität Bochum, Katholisches Klinikum Bochum, St. Josef-Hospital, Alexandrinenstr. 5, 44791 Bochum, Germany; 2 Institut für Kinderradiologie, St. Josef-Hospital, Gudrunstr. 56, 44791 Bochum, Germany; 3 Zentrum für Humangenetik der Ruhr-Universität Bochum, Universitätsstr. 150, 44801 Bochum, Germany

**Keywords:** Canavan disease, Aspartoacylase deficiency, Leukodystrophy with megalencephaly, Cerebral ultrasound, Multicystic changes in white matter

## Abstract

Canavan disease (CD; MIM 271,900) or spongy degeneration of the central nervous system (CNS) is a lethal, rare autosomal recessive leukodystrophy, first described in 1931 (Canavan in Arch Neurol Psychiatry 25: 299–308, 1931). The clinical presentation includes severe neurologic impairment and macrocephaly with onset of symptoms at the age of 3–5 months. Biochemical and genetic fundamentals of the disease are elucidated. Imaging diagnosis is principally based on MRI with important role of MR spectroscopy. We report the cerebral sonographic findings in a severely affected infant with CD: Diffuse hyperechogenicity and small multicystic changes of white matter as well as an inverted pattern of echogenicity between cortical gray and subcortical white matter. These findings are compared to to the few cases found in literature and to normal ultrasound examples. Finally, ultrasound and MRI imaging findings are correlated.

## Introduction

CD is a rare autosomal recessive leukodystrophy due to mutations in the *ASPA* gene (17p13.2) encoding the enzyme aspartoacylase that catalyzes the conversion of acetylaspartic acid (NAA) to aspartate and acetate [2]. Pathophysiology of CD is linked to increased NAA concentrations and reduced acetyl-CoA availibility, resulting in disruption of the oligodendrocyte-axon interface correlating with intramyelinic vacuoles, edema and myelin loss. Axonal fibers are preserved. NAA is part of a tri-cellular metabolic cycle in CNS involving neurons, oligodendrocytes, and astrocytes [3].

Clinical onset of the disease is regularly in infancy although there are juvenile variants [4]. Presenting symptoms of CD are poor visual contact, poor head control, weak cry and suck, progressive macrocephaly, and developmental delay with muscular hypotonia. Seizures increase in frequency over time. In severe CD, life expectancy is reduced with average survival until 10 years of life, resulting in a chronic vegetative state with autonomic crises [4]. CD is presently incurable. Treatment is symptomatic and supportive.

CD affects all ethnic groups, but occurs with greater frequency in individuals of Ashkenazi Jewish descent, where a carrier rate of 1:40–58 is estimated [5]. Many mutations have been identified. Depending on the genotype, clinical course may be milder with lesser imaging findings in neuroimaging [6].

When both parents are carriers of a CD gene, prenatal DNA diagnosis can be performed by chorionic villus sampling (CVS) or amniocentesis [7].

Diagnostic criteria are diffuse leukodystrophy and diffusion restriction in neuroimaging studies [8], elevated acetylaspartic acid in the CSF and urine [2] as well as a NAA peak in MR spectroscopy [9]

Concerning ultrasound (US) diagnosis of CD there are only few case reports in the literature from 1986 to 2014 which report white matter hyperechogenicity [10–13] as well as changes of cerebral surface [11].

We report a case of CD with additional US imaging findings encompassing isolated small cystic changes of white matter, previously not described in US literature but reported in isolated MRI studies.

## Case presentation

A 7.5 months-old female infant of non-consanguine Syrian parents presented with progressive macrocephaly, truncal hypotonia interrupted by tonic spasms of the head, and horizontal eye movements. Oral feeding was still possible.

It was delivered spontaneously at 40 weeks of gestation with a weight of 4.020 g, a height of 53 cm and a head circumference of 35 cm (50th perc.). Birth and perinatal development were uneventful.

On physical examination, head circumference had passed gradually to 46.5 cm (0.5 cm > 97th perc.) while body weight and length were within normal limits (25th–50th percentile), 7.690 g and 68 cm respectively.

**Fig. 1:**
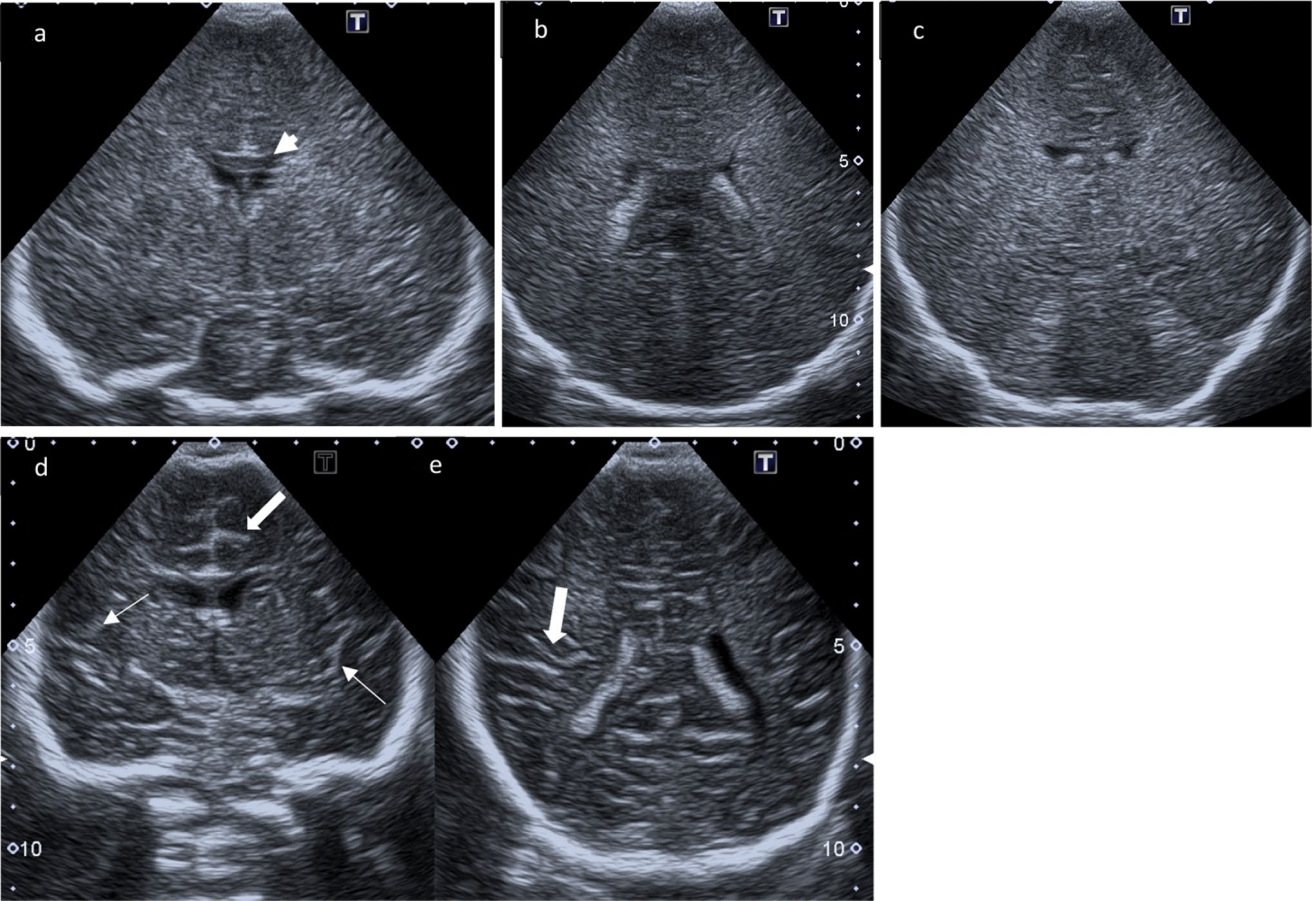
Coronal ultrasound scans. **a**–**c** Coronal scans through the foramen of Monro, the choroid plexus, and the posterior aspect of 3rd ventricle above the tentorium: generalized increased echogenicity of brain parenchyma associated with obliteration of the gyral-sulcal interfaces and narrowing of interhemispheric fissure; the Corpus callosum (arrowhead) remains hypoechoic. **d**, **e** Comparison to normal age-matched US: Coronal scans through the foramen of Monro and through the choroid plexus respectively with normal demarcation of Sylvian fissure (small arrows) and gyral-sulcal interfaces (large arrows)

**Fig. 2:**
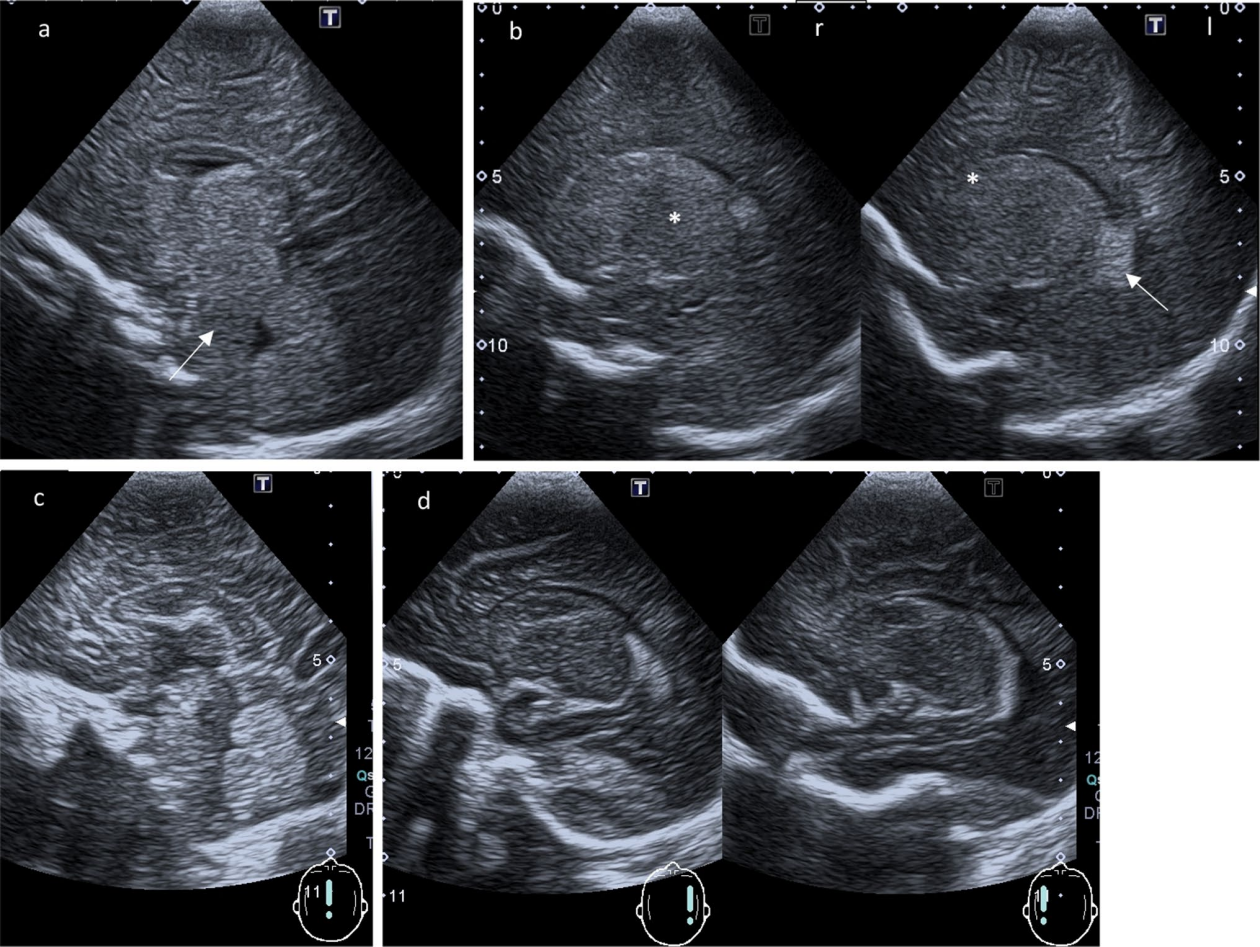
(Para-)sagittal ultrasound scans. **a** Midline sagittal plane: Increased echogenicity anterior to the fourth ventricle of pons and medulla (arrow). Third ventricle with edematous echogenic massa intermedia. **b** Parasagittal planes through the body of the lateral ventricles (right r and left l): Choroid plexus (arrow) remain highly echogenic in contrast to the normal echogenicity of thalamus (* b, r) and nucleus caudatus (* b, l). **c**, **d** Comparison to a normal age-matched child: Midline sagittal plane respectively parasagittal planes through the body of the lateral ventricles

**Fig. 3:**
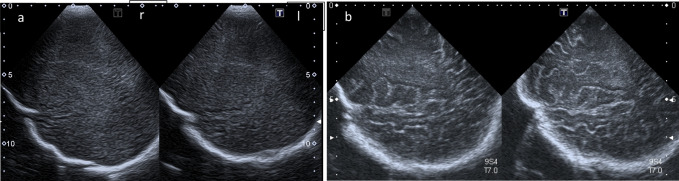
Parasagittal scans far lateral (right r and left l to the ventricles). **a** Peripheral brain parenchyma without individualization of Sylvian fissure and cerebral convolutions. The island of Reil failed to be visualized unlike in a normal scan (**b**)

**Fig. 4:**
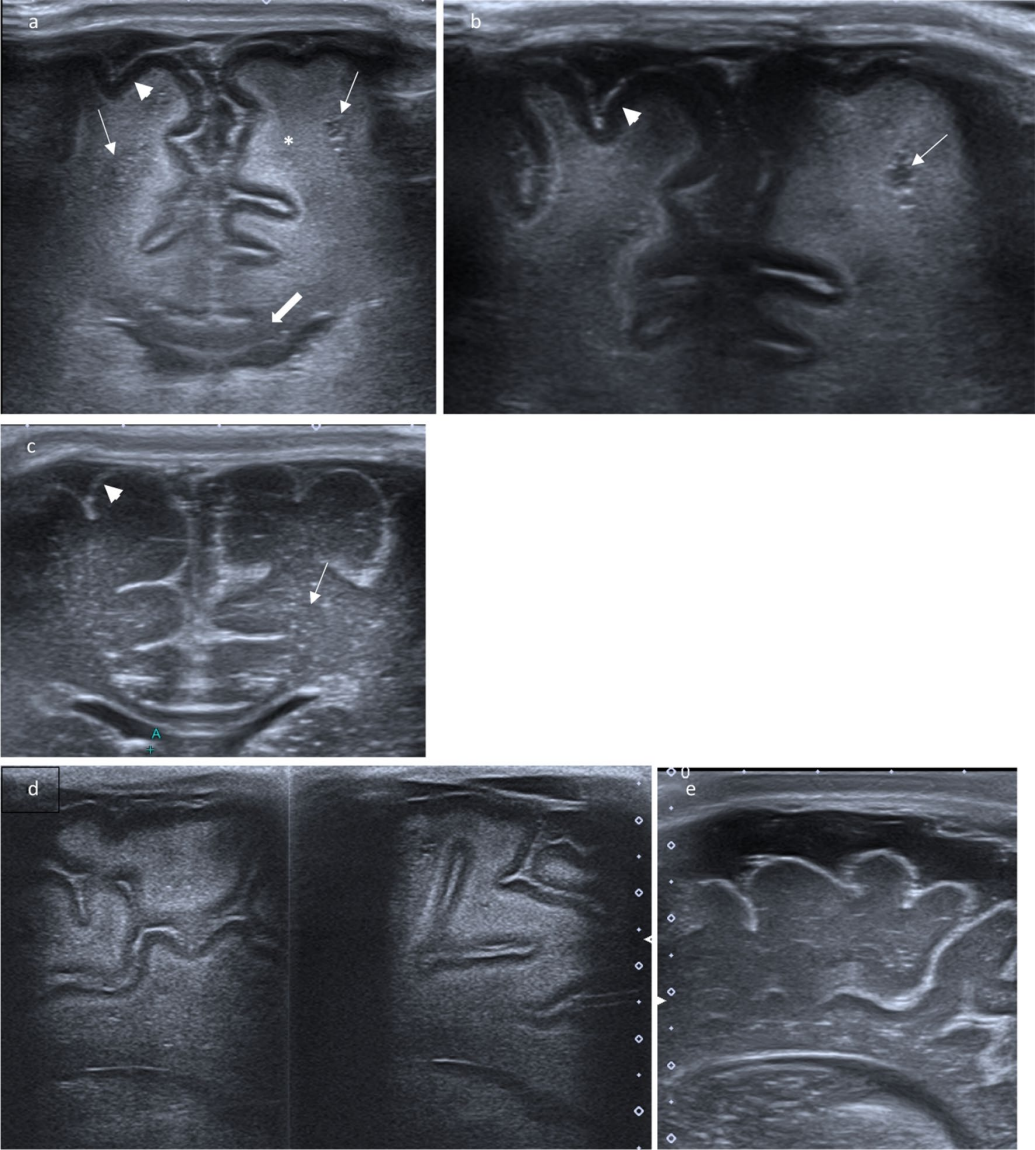
High resolution linear transducer. **a** Coronar plane: Intense hyperechoic pial demarcation. Reversal of white and gray matter echogenicity: white matter with markedly increased echogenicity (*) and a very homogeneous fine texture interrupted by small cysts (arrows); Corpus callosum remains hypoechoic (large arrow); Cortical gray matter presents as a large ribbon with low echogenicity (arrowhead). **b** White matter cysts are zoomed (arrow). **c** Coronar scan in a normal age-matched infant: Fine hyperechogenic cortical ribbon (arrowhead) and hypoechogenic white matter (small arrow). Parasagittal plane in Canavan disease (**d**) and equivalent normal ultrasound example (**e**) in an age-matched infant with benign enlargement of subarachnoidal spaces (BESS)

**Fig. 5:**
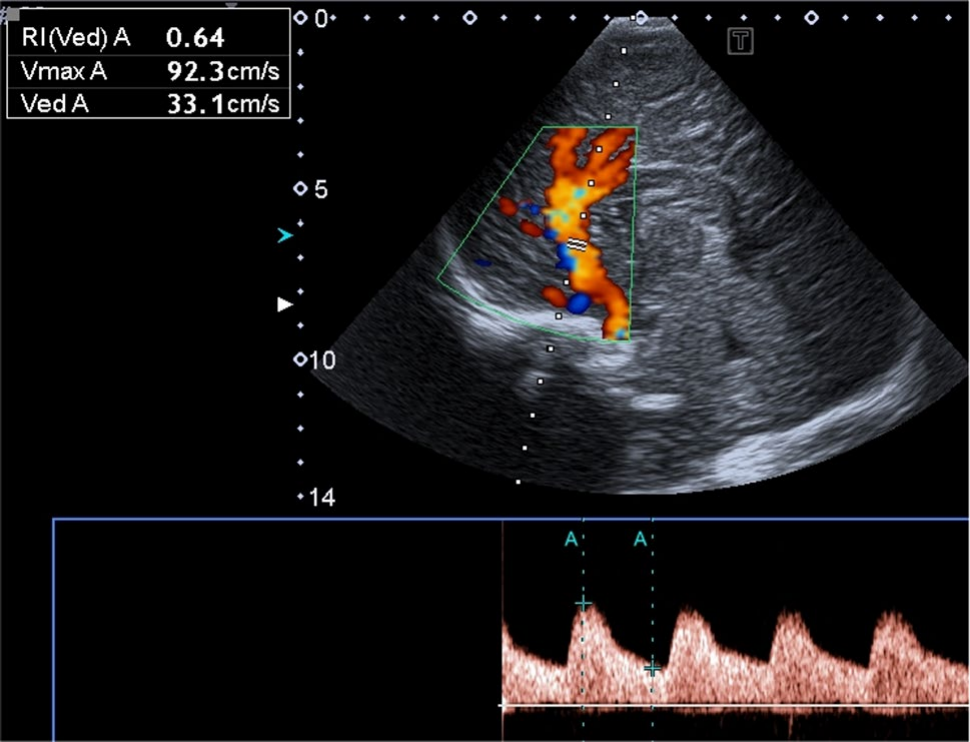
Doppler flow pattern of the major arteries show normal velocities

Cranial US (Canon Xario 200G with sector transducer (Canon, PSU-30BT 5S2, 2-5 Mhz) and linear transducer (Cannon PLU-1005BT 14L5, 5-14 Mhz)) showed general diffuse hyperechogenicity (Figs. 1, 2 and 3) of white matter including subcortical areas, blurred cerebral sulci and Sylvian fissure evocative of brain edema. A very homogeneous fine echotexture was interrupted by bilateral isolated small cysts (Fig. 4). Hyperechogenicity particularly involved the brainstem (Fig. 2a). Corpus callosum remained hypoechoic. Ventricles and pericerebral spaces were not enlarged; there were no signs of brain atrophy or intracranial hypertension (Fig. 5).

High resolution ultrasound imaging of the brain surface differed from normal findings by a reversal of the normal pattern of cortical and white matter echogenicity (Fig. 4): the cerebral cortex presenting as a hypoechoic ribbon surrounding the hyperechoic subcortical white matter and being covered by a thin hyperechoic pia mater.

**Fig. 6:**
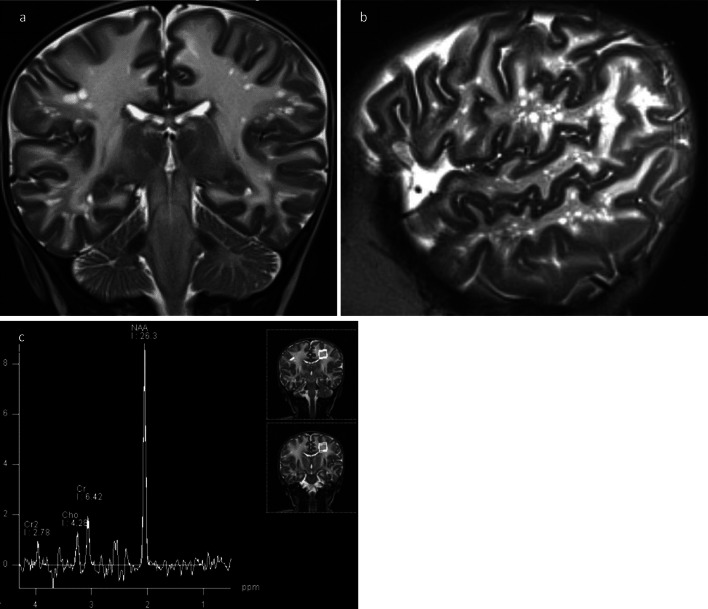
MRI. **a** T2-weighted coronar plane: hyperintense marrow areas involving subcortical and periventricular white matter accompanied with small cystic changes. **b** Spongy or “cribriform” appearance of peripheral white matter in T2-weighted sagittal plane. **c** MR spectroscopy showed markedly elevated NAA peak and NAA-creatine ratio, pathognomic for Canavan disease

MR imaging showed extended bilateral leukodystrophy of supra- and infratentorial white matter (Fig. 6a) reaching the subcortical areas and including basal ganglia with diffusion restriction on DWI. In addition, multiple bilateral small round cysts < 1 cm were visible within the lobar and subcortical white matter especially in frontal and parieto-occipital regions Fig. 6b. MR spectroscopy noted marked elevation of NAA (Fig. 6c).

Urine excretion of NAA was raised significantly above normal values with 1338 mmol/mol creatinine (*N*: < 30).

Molecular analysis of the *ASPA*-gene revealed compound heterozygoty for mutations in exon 4: c.634 + 1G > T p.? (maternally inherited) and in exon 6: c.815 T > C p.(Leu272Pro) (paternally inherited), known to be associated with CD [14, 15].

## Discussion

CD is a devastating disorder involving both white and gray matter of CNS [16] resulting in severe and progressive neurological impairment as well as macrocephaly in early infancy. US as the first line imaging technology in newborns will be applied in this setting, although the imaging diagnosis of this disease is based on MRI with proof of NAA-peak in MR spectroscopy (Fig. 6c).

The pathognomonic biochemical marker for CD is NAA accumulation in blood, urine, CSF and amniotic fluid. The role of NAA accumulation in the pathogenesis of progressive myelin degeneration in CD has been the subject of multiple investigations and theories [17, 18] well summarized by several authors [19]. NAA and particularly ASPA are believed to play a role in the molecular efflux water pump system to the extracellular fluid, obeying to a transport gradient between two juxtaposed anabolic (neurons) and catabolic (oligodendrocytes) compartments. Lack of ASPA activity leads to abnormally high osmolar levels in the periaxonal space as well as to vacuolar spongiform destruction of gray matter and existing myelin [20]. The water content of the white matter is dramatically increased [8].

This explains why the spongiform degeneration of the brain is ongoing with progressive enlargement of head circumference as macrocephaly with CD is due to megalencephaly with elevated cerebral weight and volume.

Initial edematous swelling of white matter will change to brain atrophy with ventriculomegaly in advanced stage of disease.

The  demyelination / dysmyelination theory of CD considers acetate deficiency responsible for incomplete and loose binding of myelin layers, leading to vacuoles in interstitial space.

High concentrations of NAA in brain are also thought to be toxic (oxidative stress theory) especially when linked to concurrent ASPA deficiency as shown in animal studies.

In imaging and in histology studies, the worst effect of myelin damage is at the junction of gray and white matter (subcortical arcuate “U”-fibers). Neuroimaging findings progress over time and show a centripetal spread to the central white matter. Corpus callosum and internal capsules are spared unlike white matter of brain stem and cerebellum; globus pallidus and thalamus are typically involved.

As demonstrated in previous case reports on CD [11, 12], the typical changes of the brain surface with reversal of white and gray matter echogenicity and a highly echogenic pial demarcation (Fig. 5) are well understandable considering the pathophysiology mentioned above.

Of course, transfontanellar US is not the optimal imaging approach  [16, 21, 22] to define leukodystrophy. Nevertheless, US will be applied as first line imaging method in macrocephaly. US features of leukodystrophies are described in few articles about Aicardi-Goutières syndrome (leukodystrophy with calcifications) [23] or even Canavan [10–13] and Alexander’s disease (fibrinoid leukodystrophy) [24], both leukodystrophies with megalencephaly. The hall mark of in US imaging findings in leucodystrophies is white matter hyperechogenicity. It is noted due to multiple interfaces at the level of vessels and  loss of nerve fibers myelin sheaths resulting in a higher echogenictiy of the brain tissue.

In this case of CD, we also observed generalized increased echogenicity of brain parenchyma, including brain stem (Fig. 2a), poorly defined gyral-sulcal interfaces and slit-like ventricles (Fig. 1). These findings are quite similar to those encountered in brain edema in the context of neonatal diffuse hypoxic-ischemic injury, but without obvious signs of intracranial hypertension (Fig. 5).

Small cystic changes within the white matter have not yet been reported in US assessment of CD. This finding reflects the spongiform vacuolation generally observed in histology [25], but amazingly rarely described in imaging of CD, even in MRI. In literature on pediatric neuroimaging [16, 21] this specific imaging finding is not mentioned. Only three publications [26–28] report about a honeycomb, spongy or cribriform imaging pattern of white matter in CD. Another publication [29] reports similar findings with developing of cysts in later stages of CD after the first year of life. The small cysts encountered in our study differ clearly from the radiant and linear topography of dilated perivascular spaces of Virchow-Robin (Fig. 6a, b) and are also different from the large subcortical cysts in the fronto-parietal and temporal regions found in megalencephalic leukoencephalopathy with subcortical cysts (MLC—van der Knaap disease). Therefore, we speculate that they are more representative of the giant intramyelinic vacuoles in CD, as mentioned in a recent article by Bath [28].

The imaging finding of a cystic pattern of white matter may be associated with novel mutations in the *ASPA* gene going on with a severe clinical course [28]. Further studies are necessary to elucidate why certain patients demonstrate these cysts whilst others do not present this imaging finding. Perhaps these neuropathologic changes may demand a longer period of time before being obvious in imaging studies. Furthermore, they may be age and/or genotype dependant.

## Conclusions

US imaging findings in CD reflect the pathological mechanisms mentioned above. Apart from the diffuse hyperechogenicity of brain parenchyma, as found in many leukodystrophies, other discriminative US markers for CD are the reversal of echogenicity between subcortical white and cortical gray matter, especially in combination with small cystic changes in white matter, rarely reported in imaging of CD but possibly detectable in US as demonstrated here.

## References

[1] Canavan M (1931). Schilder’s encephalitis periaxialis diffusa. Arch Neurol Psychiatry.

[2] Matalon R, Michals K, Sebesta D (1988). Aspartoacylase deficiency and N-acetylaspartic aciduria in patients with Canavan disease. Am J Med Genet.

[3] Baslow MH, Guilfoyle DN (2014). A breakthrough in understanding the nature of Canavan disease, a human spongyform leucodystrophy due to inborn errors in the gene encoding for aspartoacylase. Brain Disord Ther.

[4] Traeger EC, Rapin I (1998). The clinical course of Canavan disease. Pediatr Neurol.

[5] Feigenbaum A, Moore R, Clarke J (2004). Canavan disease: carrier-frequency determination in the Ashkenazi Jewish population and development of a novel molecular diagnostic assay. Am J Med Genet.

[6] Mendes MI, Smith DEC, Pop A (2017). Clinically distinct phenotypes of Canavan disease correlate with residual aspartoacylase enzyme activity. Hum Mutat.

[7] Matalon R, Kaul R, Gao GP, Michals K, Gray RGF, Bennett-Briton S, Norman A, Smith M, Jakobs C (1995). Prenatal diagnosis for Canavan disease: the use of DNA markers. J Inher Metab Dis.

[8] St M, Nelson GR, Longo N (2016). Cytotoxic edema and diffusion restriction as an early pathoradiologic marker in Canavan disease: case report and review of the literature. Orphanet J Rare Dis.

[9] Janson GG, McPhee SW, Francis J (2006). Natural history of Canavan disease revealed by proton magnetic resonance spectroscopy (1/H-MRS) and diffusion-weighted MRI. Neuropediatrics.

[10] Patel PJ, Kolawole TM, Mahdi AH, Wright EA (1986). Sonographic and computed tomographic findings in Canavan’s disease. Br J Radiol.

[11] Bührer C, Bassir C, von Moers A, Sperner J, Michael T, Scheffner D, Kaufmann HJ (1993). Cranial ultrasound findings in aspartoacylase deficiency (Canavan disease). Pediatr Radiol.

[12] Breitbach-Faller N, Schrader K, Rating D, Wunsch R (2003). Ultrasound findings in follow-up Investigations in a case of Aspartoacylase deficiency (Canavan disease). Neuropediatrics.

[13] Drera B, Poggiani C (2004). Brain ultrasound in Canavan disease. J Ultrasound.

[14] Rady PL, Penzien JM, Vargas T, Tyring SK, Matalon R (2000). Novel splice site mutation of aspartoacylase gene in a Turkish patient with Canavan disease. Europ J Paed Neurol.

[15] Zeng BJ, Moffett JR, Tieman SB, Weinberger DR, Coyle JT, Namboodiri MA (2006). Mutation analysis of the aspartoacylase gene in non-Jewish patients with Canavan disease. N-acetylaspartate: a unique neuronal molecule in the central nervous system.

[16] Barkovich AJ, Raybaud C, James BA (2018). Metabolic disorders that affect both gray and white matter. Pediatric neuroimaging.

[17] Taylor DLDS (1995). Investigation into the role of N-acetylaspartate in cerebral osmoregulation. J Neurochim.

[18] Baslow MH (1999). Molecular water pumps and the aetiology of Canavan disease: a case of sorcerer’s apprentice. J Inherit Metab Dis.

[19] Ahmed SS, Gao G (2014). Making the white matter matters: progress in understanding Canavan’s disease and therapeutic interventions through eight decades. JIMD Rep.

[20] Appu AP, Moffett JR, Arun P (2017). Increasing N-acetylaspartate in the brain during postnatal myelination does not cause the CNS pathologies of Canavan disease. Front Mol Neurosci.

[21] Patay Z, Tortori-Donati P (2005). Metabolic disorders. Pediatric neuroradiology brain.

[22] Siegel MJ, Siegel Marilyn J (2019). Brain. Pediatric Sonography.

[23] Rossler L, Ludwig-Seibold C, Thiels Ch, Schaper J (2012). Aicardi-Goutières syndrome with emphasis on sonographic features in infancy. Pediatr Radiol.

[24] Harbord MG, LeQuesne GW (1988). Alexander’s disease : cranial ultrasound findings. Pediatr Radiol.

[25] Gambetti P, Mellman WJ, Gonatoas NK (1969). Familial spongy degeneration of the central nervous system (van Bogaert-Bertrand disease), an ultrastructural study. Acta Neuropathol.

[26] Pradhan S, Goyal G (2011). Teaching Neuro*Images*: Honeycomb appearance of the brain in a patient with Canavan disease. Neurology.

[27] Kamate M, Kabate V, Malhotra M (2016). Spongy white matter: a novel neuroimaging finding in canavan disease. Pediatr Neurol.

[28] Bhat MD, Manjunath N, Kumari R (2021). Cribriform appearance of white matter in Canavan disease associated with novel mutations of ASPA Gene. J Pediatr Genet Georg Thieme Verlag KG.

[29] Drenckhahn A, Schuelke M, Knierim E (2015). Leukodystrophy with multiple beaded periventricular cysts: unusual cranial MRIresults in Canavan disease. J Inherit Metab Dis.

